# Supplementary material for: Systematic review and meta-analysis of the effect of ABO blood group on the risk of SARS-CoV-2 infection
Source: PLoS One. 2022 Jul 28;17(7):e0271451. doi: 10.1371/journal.pone.0271451 (PMC9333251; doi:10.1371/journal.pone.0271451)
Supplement: Attachment — Submitted filename: Response to Reviewers .docx [file pone.0271451.s004.docx]

**Response to the reviewers’ comments**

Dear Editor and Reviewers

Thank you for your suggestions and comments. We have now revised the manuscript according to these suggestions/comments that led to an improved manuscript. A point-by-point response follows.

***In-house Editor comments***

*Please acknowledge that the terminology used (and, in turn, the main aim of the study) is somehow misleading: if the present meta-analysis is focused on the association between blood type and the likelihood of infection, then you should not use the expression "COVID-19 infection", as COVID-19 refers to the disease - which may arise in a subset of patients infected by SARS-CoV-2 - but "SARS-CoV-2 infection". Conversely, if the present meta-analysis is focusing on the association between blood type and the likelihood of the disease (i.e. COVID-19), the term "infection" is redundant. But, in the latter case, all the included studies assessing the presence of the virus through RT-PCR, regardless of the clinical status of the patients, should be properly re-screened, to include only those with symptomatic subjects (if we are here evaluating the likelihood of the disease onset). Finally, if we are trying to quantify the association between blood type and both infection and/or disease onset, these are clearly two separate analyses, and the studies included in each one should be properly selected*

**Thank you for this comment, we now have updated the title: *Systematic review and meta-analysis of the effect of ABO blood group on the risk of SARS-CoV-2 infection***

***Reviewer Comments:***

***Reviewer 1***

*At least a systematic review on this topic has been already published, with similar conclusions and better methodologic assessment of the included studies. Since then, no news evidence has become available. Therefore I don’ t think that presently a replication of a previous systematic review is likely to have value.*

**Although the topic has been investigated, our study is a meta-analysis. So far published meta-analyzes on the SARS-CoV-2 infection, are either limited in number, as well as in the number of studies they include (e.g. Bhattacharjee et al 2020, Wu et al 2020, Pourali et al 2020, Golinelli et al 2020, Liu et al, 2020) or the results are still controversial. Therefore, it is easy to understand, that each new study obviously adds a little stone each time and there are no useless studies in epidemiology.**

***Reviewer 2***

1. *The abstract should include the statistically significant, import effect estimates.*

**We now provided more details and added specific results in the abstract of the revised manuscript. Thank you.**

2. *“The searching time period was restricted between February 1st 2021 to March 7th 2021”. Why are these search dates so restrictive? You are only looking at studies published within 1 month of each other? This does not make sense.*

**Indeed, this was a baffling point. In fact, the searching time period included studies with a publication deadline of March 7th, 2021.**

3. *Studies were included if Covid-19 was “clinically diagnosed”. This seriously undermines the scientific usefulness of this work. Pure clinical diagnosis of Covid-19 is very difficult given the range of presentations. Confirmatory testing is almost always used. Please exclude studies that relied on “clinically diagnosed”Covid-19.*

**Thank you for your comment. All the included studies have been re-screened for the confirmation method. The Polymerase Chain Reaction (PCR), in combination to clinical diagnosis (where indicated), has been used to detect the presence of SARS-CoV-2 virus, except for one study, where the confirmation method was not mentioned.**

4. *This study misses many reports of the association of blood type and Covid-19, but it is unclear why these studies were omitted: Ellinghaus et al., Kolin et al., Zietz et al., etc.*

**According to our inclusion criteria, our analysis included only original studies (not meta-analyzes) and studies with distinct groups, regarding COVID-19 infection and ABO blood groups state (positive vs negative) in which Odds Ratio/Risk Ratio has been calculated.**

5. *The reason for excluding particular reports, along with the number of reports excluded for that particular reason, needs to be listed in Figure 1.*

**The flow diagram was based on the inclusion and exclusion criteria, mentioned in ‘’Materials and Methods’’.**

**We would like to thank the Editor and Reviewers for their suggestions and targeted comments.**
