# Supplementary material for: Systematic review and meta-analysis of the effect of ABO blood group on the risk of SARS-CoV-2 infection
Source: PLoS One. 2022 Jul 28;17(7):e0271451. doi: 10.1371/journal.pone.0271451 (PMC9333251; doi:10.1371/journal.pone.0271451)
Supplement: Attachment — Submitted filename: Response to Reviewers_R2.docx [file pone.0271451.s005.docx]

**Response to the reviewers’ comments**

Dear Editor and Reviewers

Thank you for being positive to the revised version that we submitted. There is only one suggestion raised by R4 which we decided not to follow, and we explain below why.

***Reviewers' comments:***

*Reviewer's Responses to Questions*

*Comments to the Author*

*1. If the authors have adequately addressed your comments raised in a previous round of review and you feel that this manuscript is now acceptable for publication, you may indicate that here to bypass the “Comments to the Author” section, enter your conflict of interest statement in the “Confidential to Editor” section, and submit your "Accept" recommendation.*

*Reviewer #2: All comments have been addressed*

*Reviewer #4: All comments have been addressed*

*2. Is the manuscript technically sound, and do the data support the conclusions?*

*The manuscript must describe a technically sound piece of scientific research with data that supports the conclusions. Experiments must have been conducted rigorously, with appropriate controls, replication, and sample sizes. The conclusions must be drawn appropriately based on the data presented.*

*Reviewer #2: (No Response)*

*Reviewer #4: Yes*

*3. Has the statistical analysis been performed appropriately and rigorously?*

*Reviewer #2: (No Response)*

*Reviewer #4: Yes*

*4. Have the authors made all data underlying the findings in their manuscript fully available?*

*The PLOS Data policy requires authors to make all data underlying the findings described in their manuscript fully available without restriction, with rare exception (please refer to the Data Availability Statement in the manuscript PDF file). The data should be provided as part of the manuscript or its supporting information, or deposited to a public repository. For example, in addition to summary statistics, the data points behind means, medians and variance measures should be available. If there are restrictions on publicly sharing data—e.g. participant privacy or use of data from a third party—those must be specified.*

*Reviewer #2: (No Response)*

*Reviewer #4: Yes*

*5. Is the manuscript presented in an intelligible fashion and written in standard English?*

*PLOS ONE does not copyedit accepted manuscripts, so the language in submitted articles must be clear, correct, and unambiguous. Any typographical or grammatical errors should be corrected at revision, so please note any specific errors here.*

*Reviewer #2: (No Response)*

*Reviewer #4: Yes*

***6. Review Comments to the Author***

*Please use the space provided to explain your answers to the questions above. You may also include additional comments for the author, including concerns about dual publication, research ethics, or publication ethics. (Please upload your review as an attachment if it exceeds 20,000 characters)*

*Reviewer #2: (No Response)*

*Reviewer #4: Excellent work, the only suggestion I have, is that you should add in your table, the characteristics of the confounding or adjustment variables in each study. Also, the adjustment methods used in these studies.*

**Thank you for this, but we chose not to follow this suggestion. Adjusting for potential covariates in the analysis wouldn’t be feasible because the number of studies for each level of potential confounding factors would be small thus not allowing for a proper meta-regression model. But we will certainly follow such an approach in future work and if the number of studies is sufficient for proper analysis.**

**We would like to thank the Editor and Reviewers for their suggestions and for being positive on the revised version that we submitted.**
